# Response Surface Optimization of Water-Based Drilling Mud Viscosity Using Titanium Dioxide–Graphene Oxide Nanocomposite and Sodium Alginate Biopolymer

**DOI:** 10.3390/polym18212660

**Published:** 2026-10-27

**Authors:** Jianlong Wang, Ren Wang, Muhammad Arqam Khan, Qibing Wang, Shaine Mohammadali Lalji, Jianwei Liu, Han Ren, Yingying Li, Syed Imran Ali, Nusrat Husain, Bo Liao

**Affiliations:** 1 CNPC Engineering Technology R&D Company Limited, Beijing 102206, China; 2 School of Petroleum Engineering, China University of Petroleum (East China), Qingdao 266580, China; 3 Department of Petroleum Engineering, NED University of Engineering & Technology, University Road, Karachi 75270, Pakistan; 4 Department of Electronics & Power Engineering, National University of Sciences and Technology (NUST), Islamabad 44000, Pakistan

**Keywords:** titanium dioxide, plastic viscosity, water-based mud system, apparent viscosity, graphene oxide, sodium alginate, response surface methodology

## Abstract

Drilling fluids play a critical role in efficient cuttings transport, suspension of drilled solids, hydraulic performance, and wellbore stability, making rheological control essential for safe and efficient drilling operations. However, conventional viscosifiers may have limited performance under demanding drilling conditions, motivating the development of nanocomposite–polymer systems with enhanced rheological tunability. The work in this article explores the viscosities of drilling fluid following the incorporation of a hydrothermally synthesized nanocomposite material formed through the interaction of titanium and graphene oxide, and a biopolymer, sodium alginate. A response surface methodology (RSM) approach based on concentration was employed to systematically observe the effects of these two different entities on the viscosity of the drilling fluid. During testing, 13 experimental runs were conducted to assess the influence of particle concentration on plastic viscosity and apparent viscosity. A strong correlation was established between the experimental test runs and the predictive model generated using this RSM, with R^2^ = 96.30% (adjusted R^2^ = 93.65% and predicted R^2^ = 84.13%) for plastic viscosity and R^2^ = 96.97% (adjusted R^2^ = 94.80% and predicted R^2^ = 85.04%) for apparent viscosity, and all 95% relative errors below 15%. ANOVA showed that the nanocomposite concentration was the significant parameter with *p*-value < 0.05 in both plastic viscosity and apparent viscosity, whereas the linear effect of sodium alginate was not significant as it demonstrated *p*-values > 0.05. Increasing the nanocomposite from 0.35 to 3.5 g at 0.35 g of sodium alginate raised the predicted plastic viscosity from 9.55 to 23.95 cP (+150%) and the apparent viscosity from 18.83 to 38.03 cP (+101%). The graphene oxide provides the anchoring sites to TiO_2_ nanoparticles that ultimately increase the inter-particle contact and exert an influence on both the viscosities. Additionally, in aqueous media, TiO_2_ nanoparticles hydroxylate, thus forming hydrogen bonding with graphene oxide. This interaction reduces the slippage effect of TiO_2_ nanoparticles between the fluid layers and eventually increases the friction force of the system. Furthermore, the sodium alginate adsorbs onto the large surface area of the nanomaterial, minimizing nanoparticle aggregation and forming a stable suspension, which in turn increases the viscosity. However, the influence of the biopolymer was minimal compared with the nano-dimension nanocomposite material. Overall, the findings offer valuable insights into the synergistic effects of biopolymers and nanocomposites in improving the lifting and transportation of drill cuttings, and in mitigating wellbore instability issues.

## 1. Introduction

Drilling fluids are used to drill the subsurface rock and assist in forming a passage to the oil and natural gas reservoir beneath the surface. In general, the drilling fluid is classified into three discrete types such as oil-based mud (OBM), water-based mud (WBM), and synthetic drilling mud (SDM) [1]. Most of the drilling operations around the globe are performed using water-based drilling fluids [2]. These systems are eco-friendly, simple to formulate, and cost-effective [3]. The continuous phase for the preparation of WBM can be seawater, brine solution, or fresh water. The choice of fluid for the continuous phase in drilling mud depends on the operating conditions [4]. These fluids serve numerous critical functions during drilling activity and, in practice, most of the drilling challenges can be traced back either indirectly or directly to their efficacy [5].

One of the key functions of a drilling fluid is its lifting and carrying capacity for drill cuttings [6]. These characteristics of a drilling fluid are a function of the rheology of the mud system [7]. Mud with higher viscosity typically exhibits enhanced carrying capacity or lifting ability [6,8]. However, in high-temperature and high-pressure environments or challenging formations like shale, the mud’s performance can significantly deteriorate [9,10,11,12]. To mitigate these issues, additives are incorporated into water-based mud (WBM) systems to enhance their overall performance. These additives are in the form of salts, polymers, and nanomaterials [13].

The two most important rheological properties that relate most closely to the hydraulic performance of a drilling fluid are plastic viscosity (PV) and apparent viscosity (AV). PV and AV are in relation to the friction between the suspended solids and liquid phase with overall resistance to flow; the latter is used as an indicator of the cuttings-carrying capacity. However, if the cuttings are too thin, they will not carry well, and if too thick, will increase frictional pressure losses and ECD [14,15]. Viscosity must thus be optimized within the well geometry and operating conditions to a range of acceptable values. However, polymeric viscosifiers, including xanthan gum, have strong dependence on temperature and brine composition [16], making them unsuitable for certain challenging wells. This has led to an interest in finding additives that would increase the viscosity in a controllable and tunable manner, and nanomaterial–polymer hybrids are a promising avenue.

Researchers are currently actively exploring the use of nanoparticles in mud systems to enhance the rheological properties, maintain stability of the mud systems, and address wellbore integrity issues for more efficient and safer drilling operations. However, these particles are quite vulnerable to ionic strength, temperature, and pH [17]. Furthermore, one problem associated with the nanoparticles is the formation of aggregates, which reduces the rheological properties and can greatly affect the performance of drilling fluid due to poor colloidal stability. Recent literature has shown that nanomaterials such as multi-walled carbon nanotubes (MWCNTs) [18], silica dioxide (SiO_2_) [19], titanium dioxide (TiO_2_) [20], copper oxide (CuO) [19], zinc oxide (ZnO), and iron oxide (Fe_3_O_4_) [21,22] can considerably enhance the characteristics of a drilling fluid. The integration of nanomaterials in the drilling fluid industry has significantly revolutionized the drilling activity [23,24]. Titanium dioxide (TiO_2_) nanomaterials are particularly promising among metal oxides due to their unique combination of non-toxicity, high chemical stability, electrochemical behavior, and commercial viability [25]. In recent decades, TiO_2_ nanomaterials have attracted considerable research interest because of their diverse structure and exceptional properties. However, to overcome limitations and to further improve the functionality of TiO_2_ nanomaterials, they are usually combined with carbon-based materials such as graphene oxide (GO), MWCNTs, and activated carbon. Graphene is a two-dimensional material with a hexagonal structure [26,27]. This material exhibits strong mechanical strength, unique physicochemical properties, and has a high surface-to-volume ratio [26,28,29]. The deficiencies present in graphene can be overcome by converting it into graphene oxide (GO) through oxidation [30]. GO consists of a single-layer graphite sheet functionalized with oxygen (O)-containing groups, including hydroxyl, carboxyl, carbonyl, and epoxy, which are attached to both the surface and the edges, making it suitable for a vast range of applications [31]. These features enable GO to exhibit key functional properties, making it a valuable asset across different technological fields [32,33].

Among the carbon-based additives for water-based muds, GO has attracted particular attention. GO has been reported as a high-performance fluid-loss-control additive [34], and the rheological behavior and filtration of GO-containing muds have been examined through experiments, mechanistic interpretation, and modeling [6]. Because GO carries hydroxyl, carboxyl, and epoxy groups on its sheets, it can interact with clay surfaces, polymers, and metal oxides through hydrogen bonding and electrostatic forces, and its large surface area makes it a suitable support for TiO_2_ nanoparticles, which otherwise tend to aggregate. Titania has also been combined with polymers: a TiO_2_/polyacrylamide nanocomposite has been reported to lower filtration and raise mud viscosity [20]. The GO/TiO_2_ combination itself was evaluated for its effect on the rheological, filtration, and shale swelling characteristics of water-based mud through a full factorial design [35], and a GO–rutile TiO_2_ composite was tested in seawater-based mud [31].

Biopolymers provide an alternative method as they are biodegradable, inexpensive and can be used for water-based systems. Sodium alginate (SA) is a linear anionic polysaccharide extracted from brown algae which possesses the ability to form hydrogen bonds with graphene oxide (GO), and this property has been demonstrated for SA/GO composite films [36]. The combination of GO, pure-bore and SA has been investigated using factorial analysis to understand its impact on the filtration properties of water-based muds [37]. The combination of a nanomaterial with a biopolymer is appealing as the polymer can coat the surface of the particles and prevent particle aggregation, and the particles can strengthen the polymer network. The same was confirmed for a xanthan gum–SiO_2_ mixture that produced a higher yield point, lower filtrate loss and better lubricity than a conventional water-based mud [38].

Articles from 2026 keep evolving towards engineered and hybrid nanomaterials. At 0.01–0.1 wt.%, the best overall performance for all three was achieved at 0.07 wt.% for silica, mainly affecting the rheology; for zeolite, the best performance was at 0.05 wt.%, mainly affecting the filtration; and for graphene it was 0.1 wt.%, where the aggregation caused a degradation in performance [39]. Shuheil, Alrhym et al. 2026 prepared a nanocomposite of nitrogen- and sulfur-co-doped reduced graphene oxide (N, S–rGO) using a hydrothermal method. This reduced the volume of API filtrate in a polymeric fluid to 58.8% at an optimum loading of 1000 ppm and in a high solid fluid to 61.3%, and increased the shale recovery to 20%, though higher loadings caused agglomeration [40]. Another area of research that has been studied is the use of selenium-doped nanocomposites as drilling fluid additives [41]. Table 1 summarizes these and other representative studies, together with their main findings and limitations.

As can be seen from Table 1, the majority of nanocomposite studies focus on the filter control or shale inhibition response, and report viscosity as a secondary response. Additionally, it has been observed that the study of nanomaterials and biopolymers is conducted separately and, to the best of the authors’ knowledge, the individual and interactive effects of a metal-oxide nanocomposite and a biopolymer on plastic and apparent viscosity have not been quantified using a predictive statistical model. Furthermore, several recent studies show a decrease in performance as the aggregation level is raised above an optimum value [39,40], so concentration windows need to be identified when two additives are used simultaneously. The present work aims to fill these gaps. The TiO_2_–GO nanocomposite synthesized by hydrothermal method and the sodium alginate are mixed into the water-based mud, and the plastic and apparent viscosity of the muds are investigated through the response surface methodology (RSM). Quadratic models and the ANOVA determine which additive(s) control each viscosity and how they interact and explain the trends in the interactions between GO, TiO_2_, SA, and bentonite. Thus, the novelty of this work lies in the use of a TiO_2_–GO nanocomposite as a tunable viscosity-control system along with a biopolymer, the statistically validated predictive models that relate the two concentrations with viscosity, and the mechanistic interpretation of the interactions of the nanocomposite–biopolymer system.

The current work is divided into four different sections. The first section covers the synthesis of the nanocomposite material using the hydrothermal method. In the next part, the nanocomposite was combined with a biopolymer to formulate the water-based mud. In the third section, the rheological properties of all of the studied samples were experimentally investigated using a rheometer. Finally, using the central composite design of the experiment, the influence of both the particles on different viscosities was studied. All of the testing was conducted at standard temperature and pressure conditions. Moreover, the API 13B-1 water-based drilling fluid standard was methodically followed during the experiments.

## 2. Materials and Methodology

### 2.1. Experimental Methodology and Formation of Titanium Dioxide and Graphene Oxide Nanocomposite

**Figure 1:**
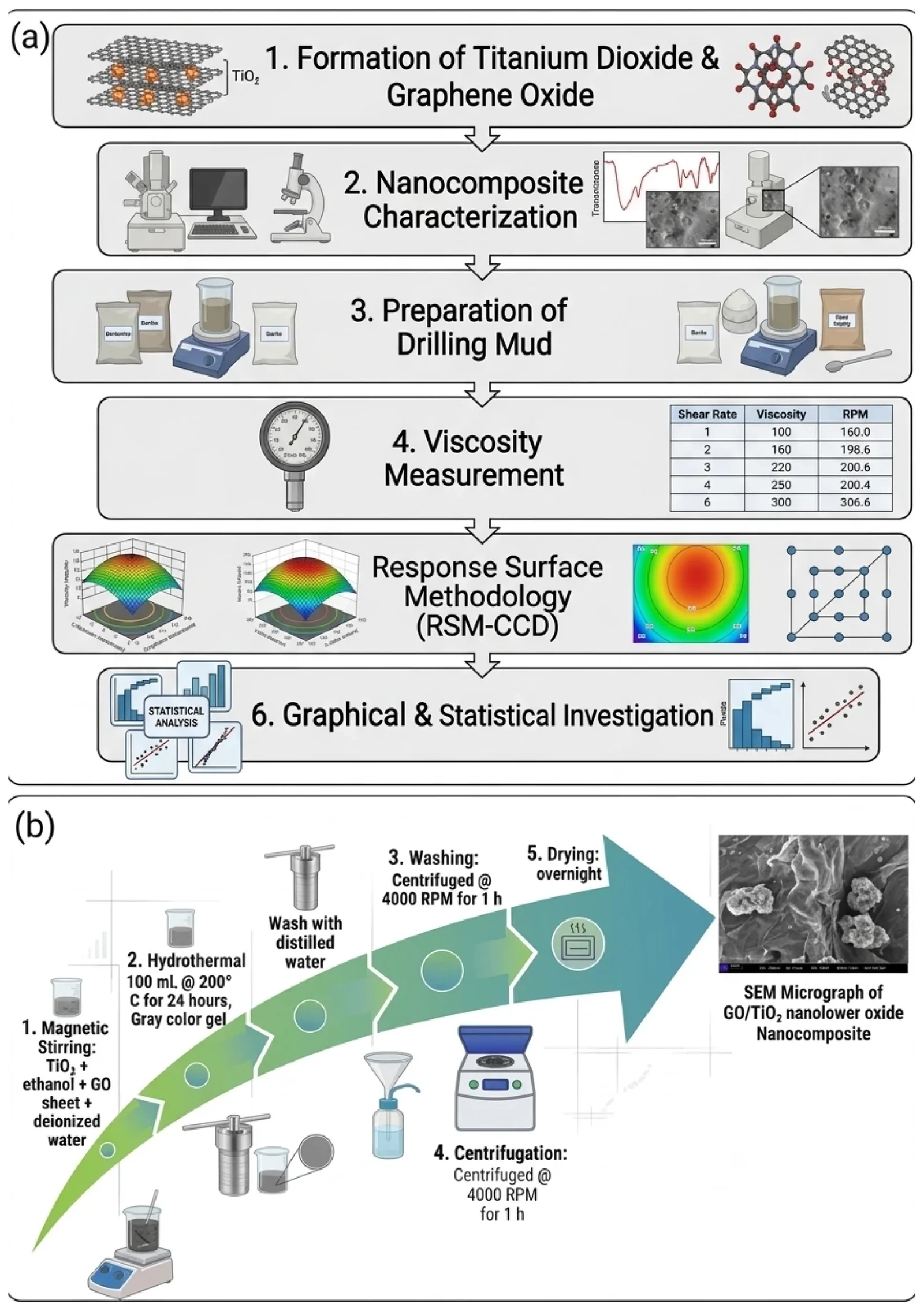
(**a**) Experimental methodology. (**b**) Detailed experimental setup used during the synthesis process.

Figure 1a shows the experimental methodology implemented in the current study. The studied particle titanium dioxide and graphene oxide nanocomposite was synthesized using the hydrothermal method, a popular approach commonly used for the synthesis of carbon-supported TiO_2_ nanoparticles due to its simplicity and effectiveness. Figure 1b shows the detailed experimental setup used during the synthesis process. The carbon material was dispersed in deionized water and sonicated for 1.5 h to prepare a homogeneous suspension. Separately, a solution of titanium (IV) isopropoxide in ethanol was prepared and added dropwise into the already prepared homogeneous solution of GO and deionized water under vigorous mixing. The resulting mixture was ultra-sonicated for 1 h to ensure uniform dispersion and improve the interaction between GO and titanium dioxide nanoparticles. The suspension was transferred to a Teflon-lined stainless-steel autoclave and was hydrothermally treated at 200 °C for 8 h. Throughout the synthesis process, the pH of the solution was maintained in the alkaline region by the addition of sodium hydroxide (NaOH). Once the reaction was complete, a gray-colored product was collected that was washed repeatedly with distilled water to remove unreacted precursor and residual impurities. The final purified product was subsequently washed with distilled water and centrifuged at 4000 rpm for 1 h before drying overnight. The resulting particle was used as a drilling fluid additive to improve the characteristics of the drilling fluid system.

### 2.2. Nanocomposite Characterization

**Figure 2:**
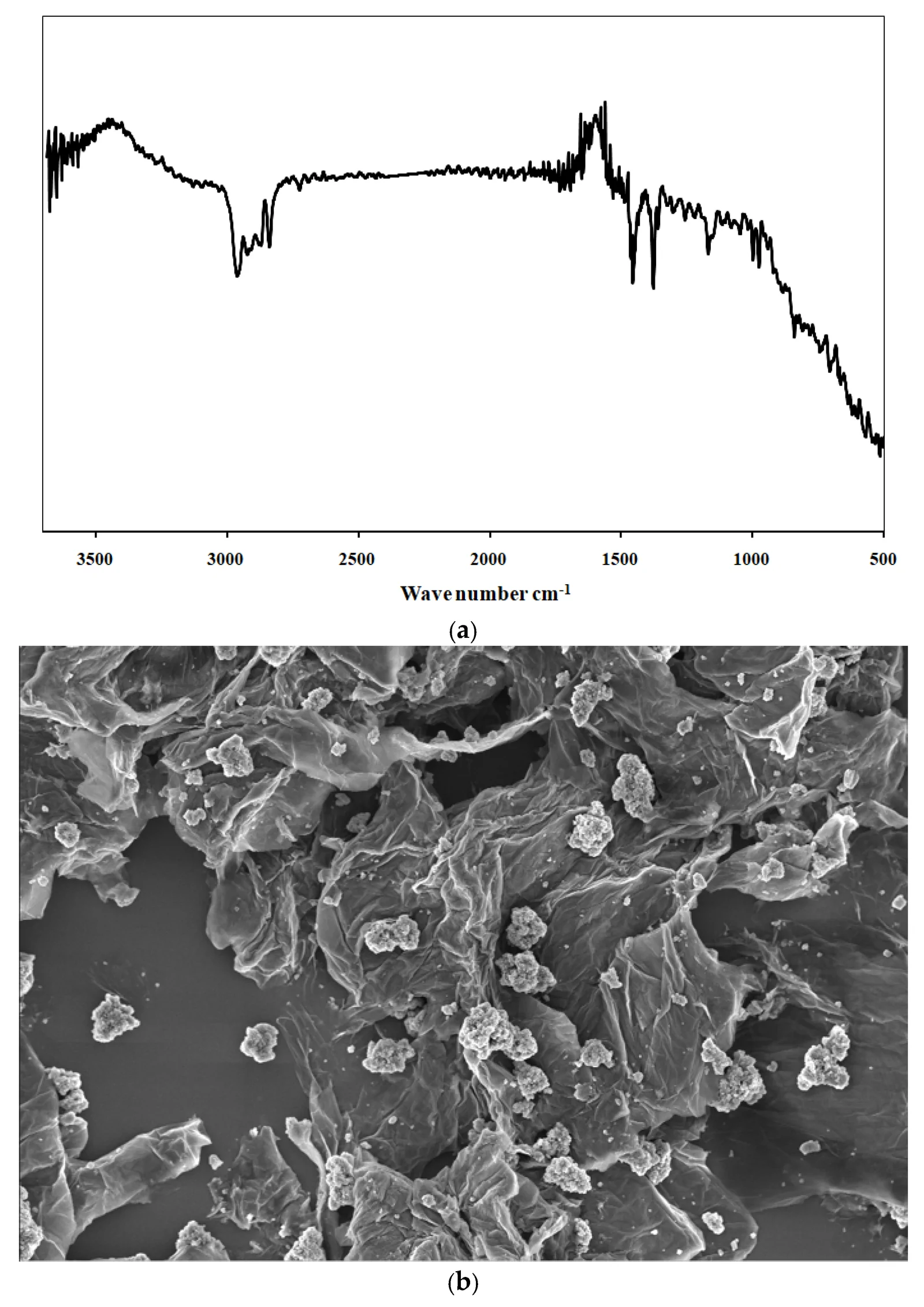
Characterization of the nanocomposite material. (**a**) FTIR spectrum and (**b**) SEM imaging.

The nanocomposite used in this study was characterized by Fourier transform infrared spectrum and scanning electron microscope imaging (SEM), as shown in Figure 2. The FTIR spectrum in Figure 2a exhibits characteristic peaks: O-H stretching at 3430.10 cm^−1^ [42], C-H symmetric and asymmetric vibrations at 2890.05 cm^−1^ and 2925.30 cm^−1^ [35], C=C stretching at 1637.56 cm^−1^, and C-OH stretching at 1375.24 cm^−1^ [43]. The broad peak between 500 and 1000 cm^−1^ corresponds to Ti-O-C and C-O-C vibrations [35]. On the other hand, the SEM imaging in Figure 2b shows the uniform distribution of clusters of TiO_2_ particles over the sheet of graphene oxide.

### 2.3. Preparation of the Drilling Mud

The study is based on the impact of synthesized particles on the viscosity of the water-based mud. The base mud consists of 302 mL of water (continuous phase), 0.3 g of sodium carbonate (for the treatment of water contamination—mixing time 5 min), 65 g bentonite (viscosifier with a specific gravity of 2.6—mixing time 10 min) and 70 g of barite (weighing agent with a specific gravity of 4.2—mixing time 10 min). As listed, the additives were added sequentially in the water and the mud was prepared using a Hamilton Beach mixer. The mud weight of the base mud was 10 ppg. The two studied particles, sodium alginate (biopolymer [3]) and the titanium dioxide and graphene oxide nanocomposite, were added according to the concentration provided by response surface methodology, with mixing times of 10 min each. The influence of these two particles on the viscosity of the mud system was observed and investigated in this article. During formulation, API standards [44] were meticulously followed. All additives, except for the nanocomposite, were sourced from a drilling company in Pakistan. During mud preparation, the additives were added in a specific sequence to minimize lump formation and increase mud performance.

### 2.4. Rationale for Using the Selected Composition of Drilling Mud

A simple bentonite–barite mud was used as the base fluid because bentonite provides the viscosity and the filter-cake-forming solids, barite provides the density, and sodium carbonate treats the hardness of the make-up water [45]. Keeping the base fluid free of other polymers, salts, and surfactants means that changes in plastic and apparent viscosity can be attributed to the two test additives and their interaction. Sodium alginate was selected as the biopolymer because it adsorbs on the negatively charged bentonite surface through hydrogen and ionic bonds and has been reported to maintain rheology and filtration better than Na-CMC and PAC-LV under salt contamination [46]. Additionally, it interacts with graphene oxide through hydrogen bonding [36] and has been combined with graphene oxide in water-based mud [37]. The TiO_2_–GO nanocomposite was selected because the GO sheets provide oxygen-containing anchoring sites and a large surface area that limit TiO_2_ aggregation, and GO/TiO_2_ materials have already been shown to modify the rheology and filtration of water-based mud [31,35].

### 2.5. Viscosity Measurement

The viscosities were evaluated following API testing protocols 13B-1 [44], with measurements taken at ambient conditions. The FANN 35 viscometer was used to measure the viscosities of the study’s samples. The viscometer used for the measurements has a dial reading accuracy of ±0.5. The viscometer data were collected at 600, 300, 200, 100, and 6 RPMs. The viscosities were calculated using the standard equations for plastic viscosity and apparent viscosity. Each experimental formulation was tested three times, and an average result is reported in the tables along with ± standard deviation.

### 2.6. Response Surface Methodology–Central Composite Design (RSM-CCD)

The central composite design (CCD) is one of the most widely utilized experimental designs in RSM for exploring the impacts of independent variables and improving response parameters. This methodology integrates axial points, factorial points and center points to estimate linear, interaction and quadratic effects so as to develop second-order polynomial models that can represent non-linear and complex relationships [47]. The variations in the position of the axial points develop three CCD configurations: face-centered (CCF), circumscribed (CCC) and inscribed (CCI). In coded design, factorial points are generally set at ±1 while axial points are typically found at ±α, based on the chosen setup [48]. The factorial points help in the estimation of main and interaction effects, while the axial and center points provide information related to quadratic effect and experimental variability respectively. The overall experimental runs are mainly associated with the quantity/levels of the factors, factorial points, axial points and center points.

### 2.7. Experimental Replication and Control of Experimental Error

Several measures were taken to limit and quantify experimental error. First, the central point of the design (1.93 g of sodium alginate and 1.93 g of nanocomposite) was replicated five times (runs 9–13), which gives an estimate of pure experimental error with four degrees of freedom and allows curvature of the response surface to be checked. Second, the 13 runs were performed in randomized order so that drift in mixing, aging of the mud, or instrument condition could not be confounded with the factor levels. Third, every sample was prepared with the same base-mud recipe, the same mixer, the same additive sequence, and the same API procedure (API RP 13B-1 2019), and all additives other than the nanocomposite came from the same supplier. Fourth, all readings were taken with the same FANN 35 viscometer at ambient conditions.

The dial-reading accuracy of the viscometer is ±0.5. Because PV = θ_600_ – θ_300_ and AV = θ_600_/2, this corresponds to an instrument uncertainty of about ±0.7 cP for PV (root sum square of two independent readings; ±1.0 cP in the worst case) and ±0.25 cP for AV.

### 2.8. Graphical and Statistical Investigation

The statistical and graphical analyses were conducted on the central composite design methodology, yielding predictive second-order equations for viscosities. The model was validated through graphical and statistical analysis. The generalized equation for the viscosities is shown in Equation (1).

(1) *Viscosity* (*cP*) = *y*_0 + *y*_1 ∗ *SA* + *y*_2 *G*/*T* + *y*_3 *SA*^2 + *y*_4 ∗ *G*/*T* ∗ *G*/*T* + *y*_5 * *SA* ∗ *G*/*T*

The response variable calculated from the model represents the viscosity of the mud systems under observation. The model’s parameters, SA (g) and G/T (g), represent the concentrations of polymeric material, in grams, of sodium alginate and nanocomposite between graphene oxide and titanium dioxide. The coefficients *y*_0 − *y*_5 in Equation (1) represent adjustable parameters that refine the response variable equation. Moreover, ANOVA analysis further evaluates the influence of the particle on the viscosity of the fluid system.

The graphical analysis was conducted using various plots, including main, interactive, contour, and surface plots. The inconsistencies between the experimental and model observations were graphically visualized through the radar and relative error plots. Additionally, both the experimental test results and model-predicted results were compared using cross-plots. All of the statistical and graphical analyses were conducted using the MINITAB 17.1 model.

## 3. Result and Discussion

### 3.1. Influence of Particles on Viscosities

**Figure 3:**
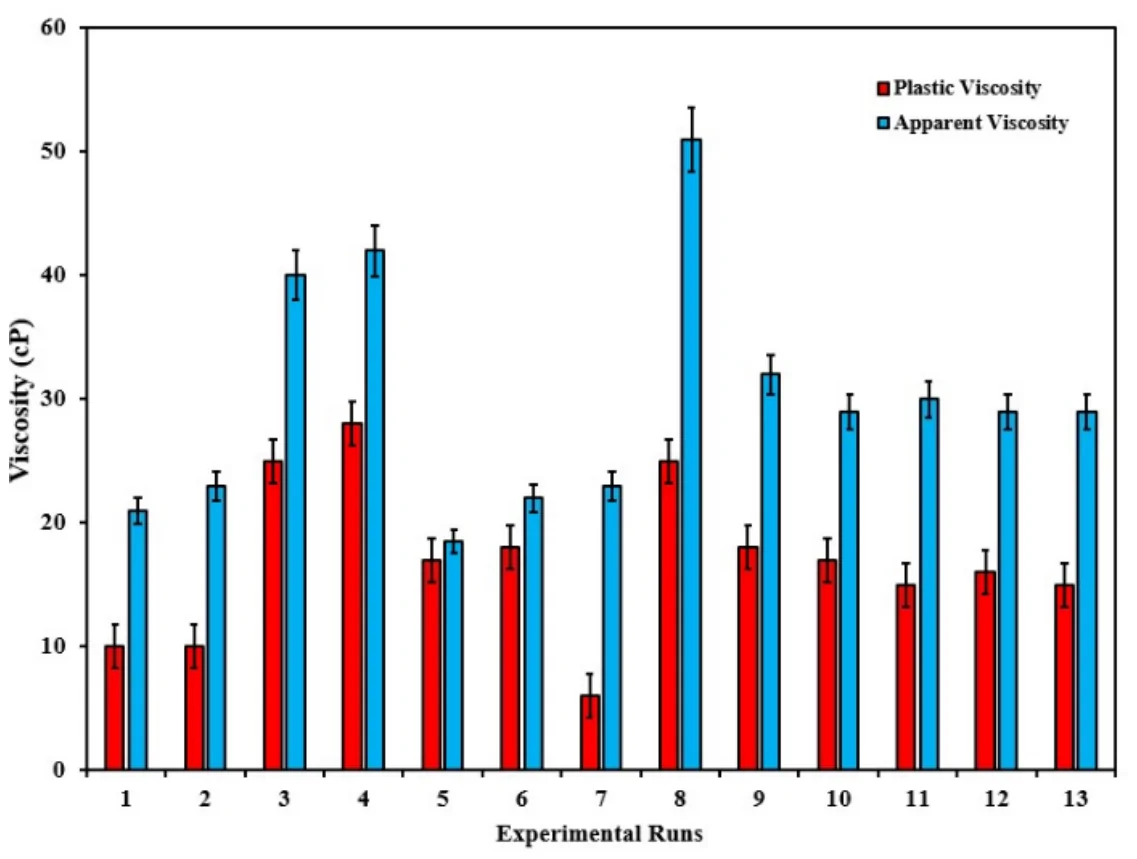
Viscosities of 13 experimental runs, obtained from the viscometer.

The movement of drilling fluid in the mud circulation system is influenced by two important viscosities: plastic viscosity and apparent viscosity. The study quantifies the viscosities of drilling mud using the viscometer test, adhering to API standard 13B-1. Table 2 outlines the central composite design matrix for sodium alginate and nanocomposite material. The initial design involves two levels of concentration of particles in the mud, 0.35 g and 3.5 g, which yields 13 experimental runs shown in Table 2. The design employs a full factorial approach, comprising factorial, axial, and center points to capture the curvature. The α-value, representing the distance between the axial and center points, is mathematically expressed as α = ${\left(2^{f}\right)}^{0.25}$. The *f* in this expression defines the number of factors under observation. In this study, α equals 1.4142, which remains relatively constant throughout the experiment. The central points from runs 9–13 in Table 2 were repeated five times, allowing for precise estimation and error reduction. The axial runs (5–8) were also conducted to capture the response surface shape effectively. All of the experimental runs were randomized, which helped in the elimination of systematic errors. The plastic and apparent viscosity for each experimental run is graphically shown in Figure 3. Plastic viscosity is a fundamental rheological property that represents the friction between the solid particles and the liquid phase, as well as the internal friction between the different liquid layers. The plastic viscosities of runs 3, 4, and 8 demonstrated higher plastic viscosity, which is mainly due to the higher solid concentration in the mud system. These samples contain a higher quantity of nanocomposite material, which increases the internal friction. These particles, because of their nano-dimension, increase internal friction within the drilling mud along with its thickening properties. Additionally, the PV results were well aligned with Smoluchowski’s modification of the Einstein equation that describes the correlation between the plastic viscosity and the size of the particle. The plastic viscosities of runs 1, 2, and 7 were on the lower side, as these samples contain the least concentration of nanocomposite materials. Apart from the nanocomposite material, the mud samples also contain sodium alginate, a long-chain polymeric material [49]. The plastic viscosity of the mud improves at a higher concentration of sodium alginate. Through cation bridging and hydrogen bonding, the anchoring groups present in the sodium alginate strongly bind with the bentonite surface present in the base mud. This strong interaction between the two entities improves the plastic viscosity of the mud. However, the improvement was slightly lower than the composite material. Furthermore, Figure 3 also illustrates the influence of both particles on apparent viscosity. This parameter plays a significant role in well cleaning and cutting transportation. According to the API guidelines, the minimum apparent viscosity for a fluid should be at least 12 cP. In the current work, the apparent viscosity of the 13 samples was greater than 12 cP. Runs 3, 4, and 8 demonstrated higher apparent viscosity than the remaining ten samples. These runs contain the maximum concentration of nanocomposite material. The thixotropic characteristics of the mud improve significantly, which ultimately improves the hole-cleaning and cutting transportation mechanism. The lowest apparent viscosity was recorded in runs 5 and 7, which contain zero weight percentage of sodium alginate and nanocomposite material, respectively.

### 3.2. Investigation of Residual and Normality for Plastic Viscosity

**Figure 4:**
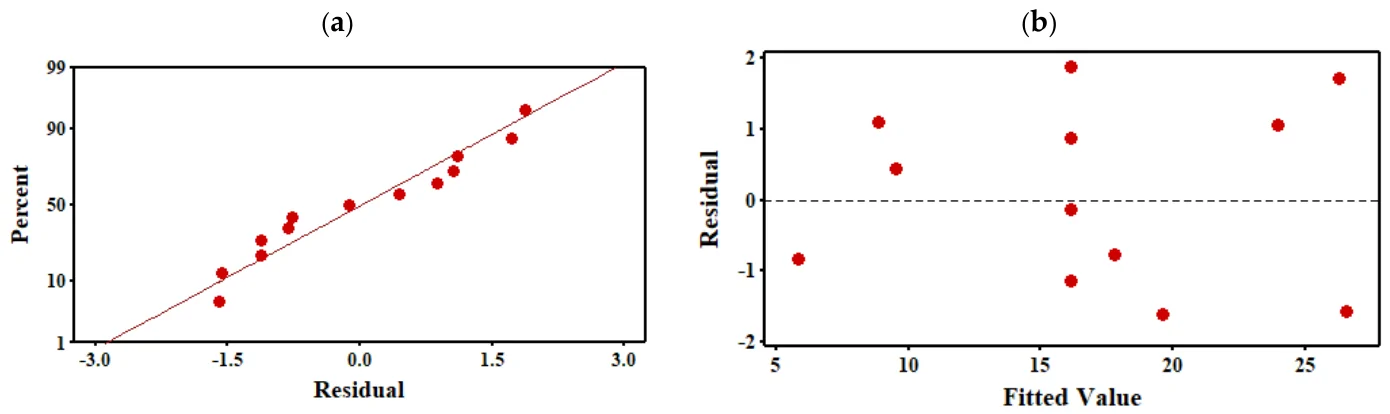

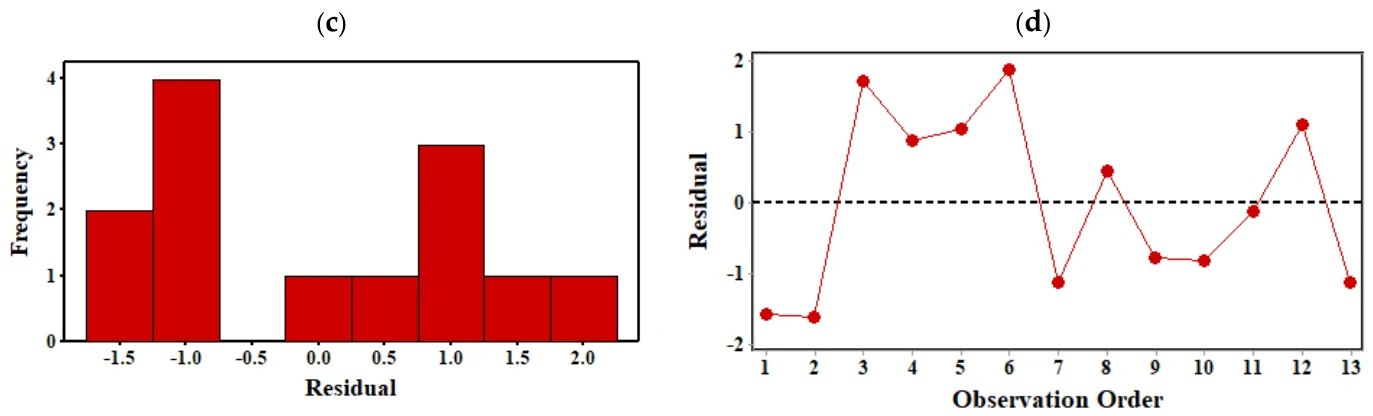
Analytic plots for plastic viscosity. (**a**) Normality plot; (**b**) residual error plot; (**c**) residual frequency bar-charts; and (**d**) residual at each run.

Figure 4 displays different analytical plots developed using MINITAB 17.1 software. These plots evaluate the performance and efficacy of the plastic viscosity model developed in this article. The normality plot in Figure 4a shows that the residual points closely follow the straight line, which indicates a normal distribution trend and a strong model performance in predicting the plastic viscosity of the mud samples. The residual errors and residual frequency plot in Figure 4b,c provide further comprehension into the model’s performance. Points overlapping the zeroth line demonstrate perfect alignment between experimental and model values. These points further indicate zero residual error between the two entities. Points away from the zeroth line indicate a discrepancy between the model and the experimental result. In the current work, six samples are near the zeroth line, showing good agreement between the experimental and model results. These experimental runs were 2, 8, 10, 11, 12, and 13. All of these runs contain a high concentration of nanocomposite material. The higher aspect ratio of the nanomaterial leads to an increase in their interaction between the material and the fluid’s components. On the other hand, experimental run 2 exhibited the largest positive residual error. This sample contains the least concentration of the nanocomposite material.

### 3.3. Influence of Particles on Plastic Viscosity Through Interaction and Main Effect Plot

**Figure 5:**
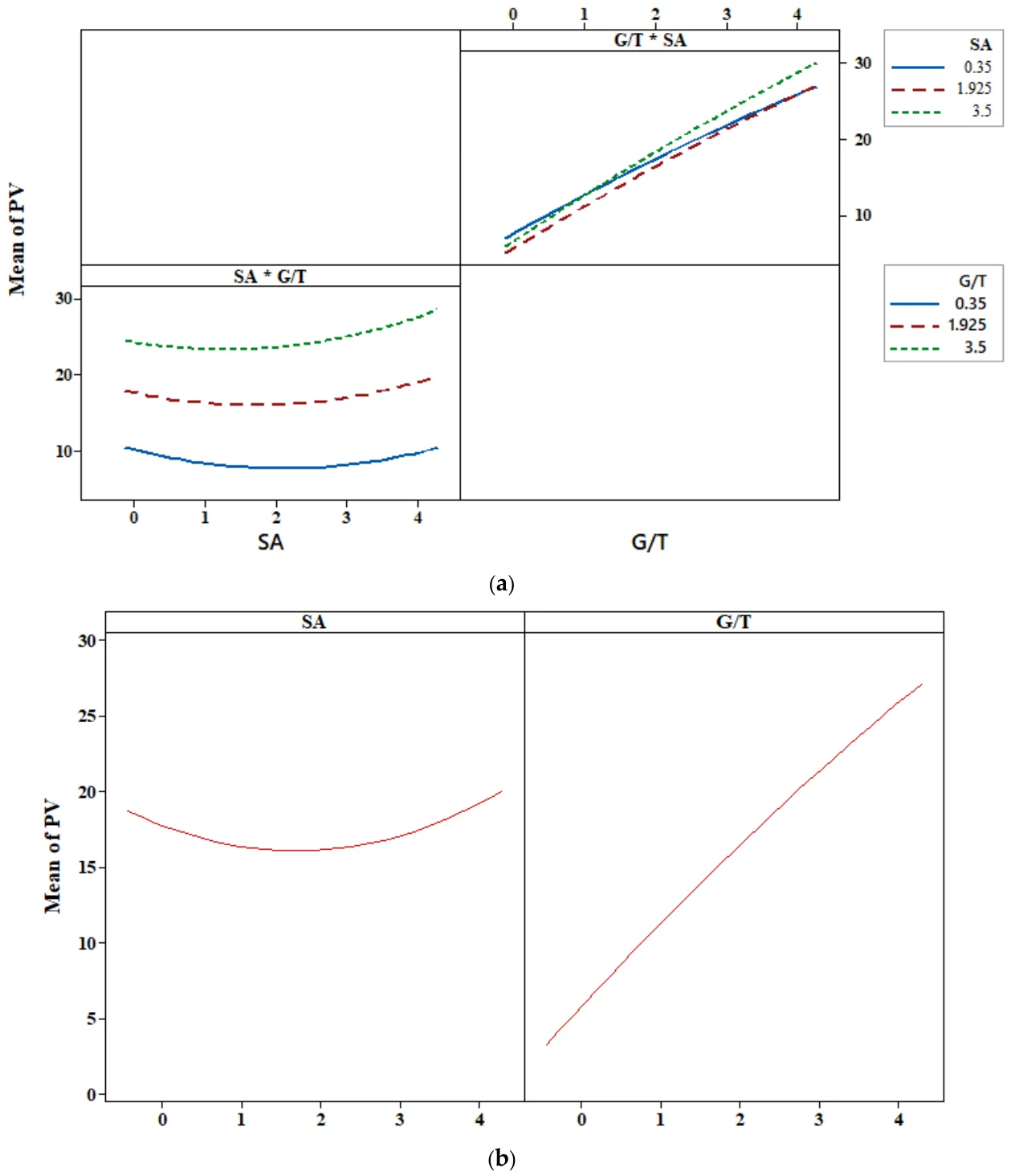
Analyzing the influence of particles on plastic viscosity using (**a**) interaction plot and (**b**) main effect plo (* means combine effect).

The individual influence of particles on the plastic viscosity was further investigated using the interaction and main effect plot. Figure 5a demonstrates the interaction matrix plot between the two materials and their influence on the response factor (plastic viscosity). A significant interaction effect is characterized when the lines representing different factors in the interaction plot remain non-parallel to one another [37]. The most impactful parameter is the nanomaterial concentration, which substantially influenced the plastic viscosity. The interaction matrix clearly demonstrates that the concentration of the nanomaterial played a significant role in influencing the plastic viscosity. The maximum impact on plastic viscosity was recorded at 3.5 g of nanoparticle concentration. The increase in viscosity is mainly due to the interaction between the oxygenated functional groups in graphene oxide and the sodium alginate polymer chain [36,50]. Figure 5b shows the individual influence of the particle on the plastic viscosity. It is evident that both the studied particles demonstrated a positive response to the plastic viscosity. An increase in the concentration ultimately increases the plastic viscosity of the drilling mud. However, the most influential parameter is the nanomaterial (composite of GO and TiO_2_). The high loading of the composite material increases the particle–particle and particle–polymer interactions, which eventually influences the plastic viscosity. Additionally, the interaction between the planar GO and the spherical TiO_2,_ as shown in Figure 2b, affects the flow behavior of the drilling mud. On the other hand, sodium alginate exhibited a limited effect on the plastic viscosity of the drilling mud. This biopolymer increases viscosity through two primary mechanisms: polymer chain entanglement and hydration. Together, these factors cause a moderate increase in the flow resistance of the drilling mud.

### 3.4. 2D Contour and 3D Surface Plot

**Figure 6:**
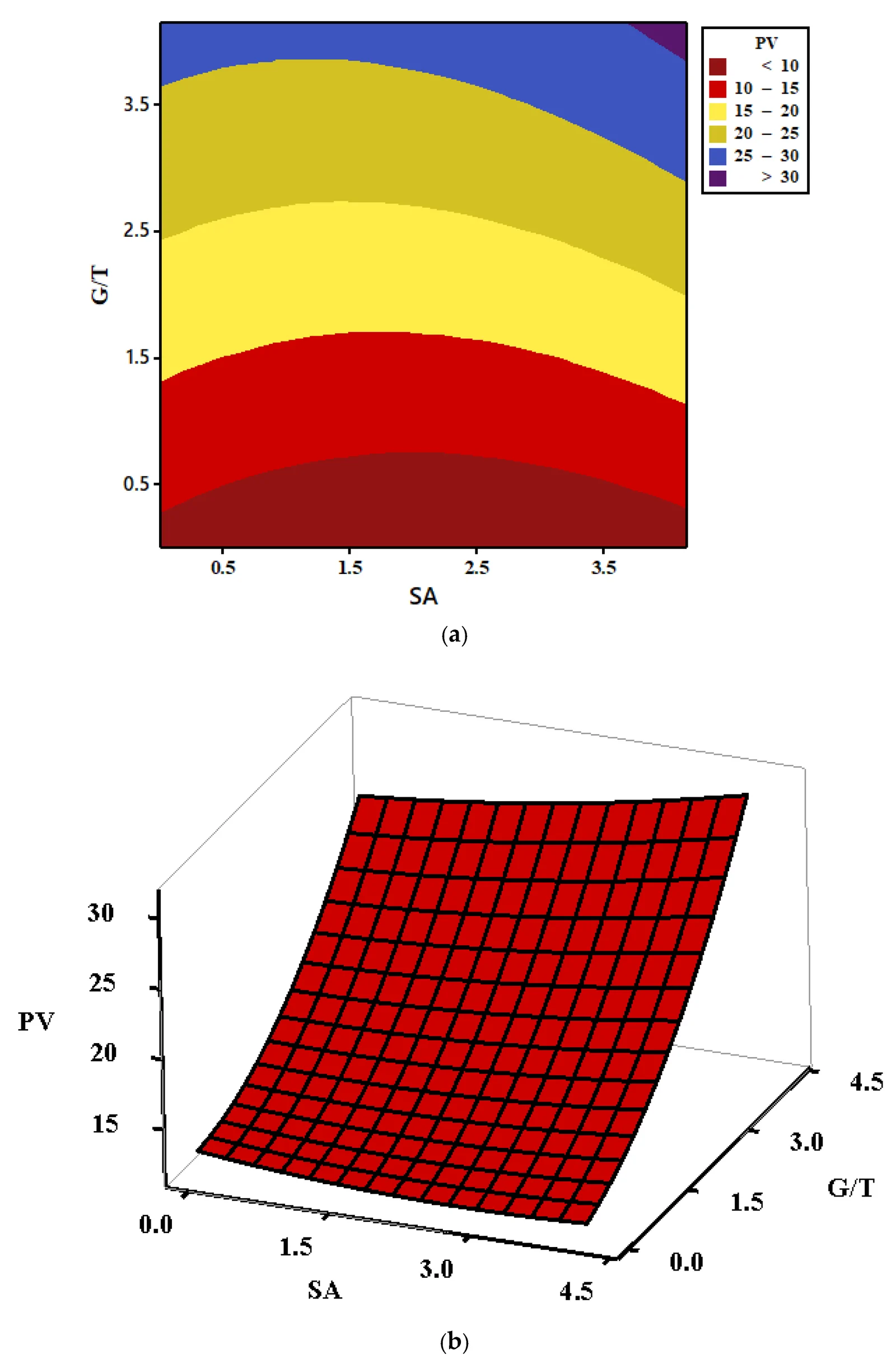
Influence of nanoparticles and sodium alginate on plastic viscosity through (**a**) contour plot and (**b**) surface plot.

Two-dimensional contour and three-dimensional surface plots are the parts of response surface plots that facilitate the visualization and analysis of interactions between the operating variables and the response parameters [51]. Figure 6a depicts the 2D contour plot, which further validates the information presented in Figure 5a. The contour plot clearly shows that the interaction between the nanocomposite and polymeric material impacts the plastic viscosity. The hydroxyl (-OH) and carboxylate groups (-COOH) enable strong communication between the nanomaterial and polymer through hydrogen bonding and electrostatic forces. This interaction gives rise to a robust network structure within the drilling fluid, which eventually increases the plastic viscosity. Furthermore, the sodium alginate adsorbs onto the large surface area of the nanomaterial, minimizing nanoparticle aggregation and forming a stable suspension, which in turn increases the plastic viscosity. Figure 6b shows a 3D surface plot that further demonstrates the influences of both the studied particles on the response variable (plastic viscosity). This plot validates the result presented in Figure 5b and clearly shows that the most significant parameter is the nanomaterial concentration, which substantially improves the plastic viscosity of the drilling mud. The high surface area and oxygen-bearing functional groups associated with the graphene oxide promote robust interaction with TiO_2_ particles. The composite tends to form a strong network structure, which ultimately enhances the plastic viscosity of the drilling mud.

### 3.5. Regression Model and Analysis of Variance

A second-order predicted plastic viscosity equation, as shown by Equation (2), was developed in this article. The parameters ‘SA’ and ‘G/T’ represent the concentration (grams) of the polymeric material, sodium alginate, and the nanocomposite material: graphene oxide/titanium dioxide. The constants in Equation (2) are the adjustable parameters that show the best-fitted equation for the plastic viscosity. The result calculated from the model equation was statistically tested using the ANOVA analysis. Table 3 shows the result of the analysis of variance for the plastic viscosity model. The adequacy and quality of the predicted model were assessed using this statistical parameter. In the current study, the model demonstrated a statistically significant result, with a *p*-value < 0.05, which clearly indicates that the null hypothesis was rejected and that there is some statistical significance between the model and the experimental plastic viscosity result. Moreover, the nanocomposite material emerged as the most influential parameter, demonstrating the highest statistical significance (*p*-value < 0.05) when compared with sodium alginate. The graphene oxide provides the anchoring sites to TiO_2_ nanoparticles that ultimately increase the inter-particle contact and exert an influence on the plastic viscosity. Additionally, in aqueous media, TiO_2_ nanoparticles hydroxylate, thus forming hydrogen bonding with the graphene oxide. This interaction reduces the slippage effect of TiO_2_ nanoparticles between the fluid layers and eventually increases the plastic viscosity of the system. Additionally, the PV model developed in this study proves to be significant, with a *p*-value < 0.05 as shown in Table 3, while the lack of fit remained statistically insignificant, which clearly indicates that the newly developed regression model effectively describes PV experimental values. Furthermore, the coefficient of determination R^2^ = 96.30% shows that almost all of the experimental datasets can be properly explained through this model. The close agreement between R^2^ adjusted = 93.65% and R^2^ predicted = 84.13% further indicates the exceptional predictive capability without any indication of model overfitting.

(2) **Plastic Viscosity (cP)** = 8.58 − 2.56 ∗ SA + 5.08 ∗ G/T + 0.582 ∗ SA ∗ SA − 0.160 ∗ G/T ∗ G/T + 0.302 ∗ SA ∗ G/T

### 3.6. Graphical Validation of the Developed Model

**Figure 7:**
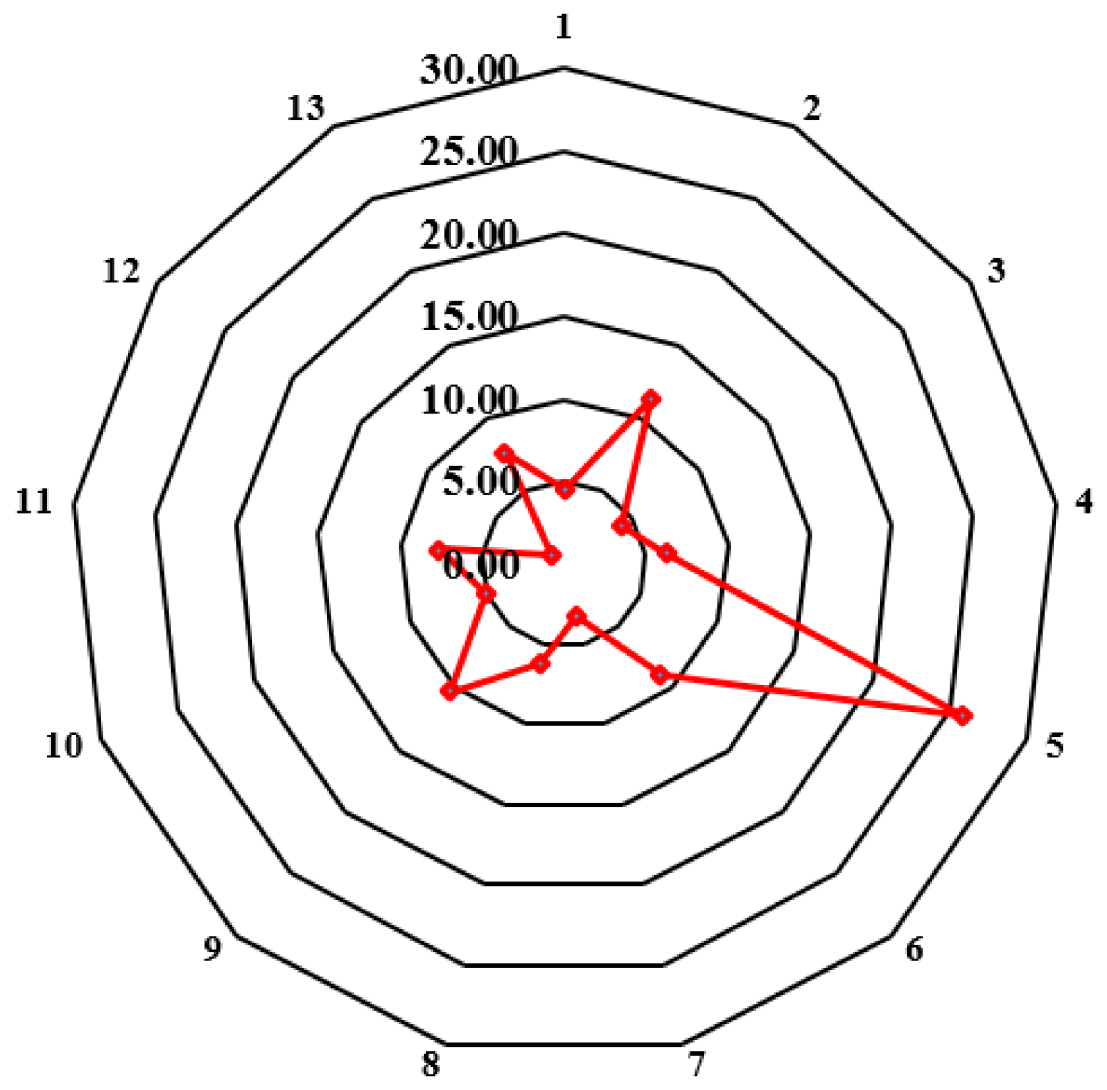
Graphical validation of the plastic viscosity regression model through relative error radar plot.

The graphical validation of the plastic viscosity regression model is shown in Figure 7. Figure 7 shows the relative errors radar plot against each experimental run. The maximum error of 10% was recorded in this radar plot against experimental run 9. All of the other runs demonstrated the error below 10%. This clearly indicates the efficacy of the developed model in predicting the plastic viscosity of the drilling mud. Table 4 shows the model-predicted plastic viscosities and the corresponding relative error percentages. It is evident that the relative error percentages of 12 out of 13 experimental runs are below 15%. Moreover, the maximum error was recorded in sample 5, which contains only nanocomposite material.

### 3.7. Investigation of Residual and Normality for Apparent Viscosity

**Figure 8:**
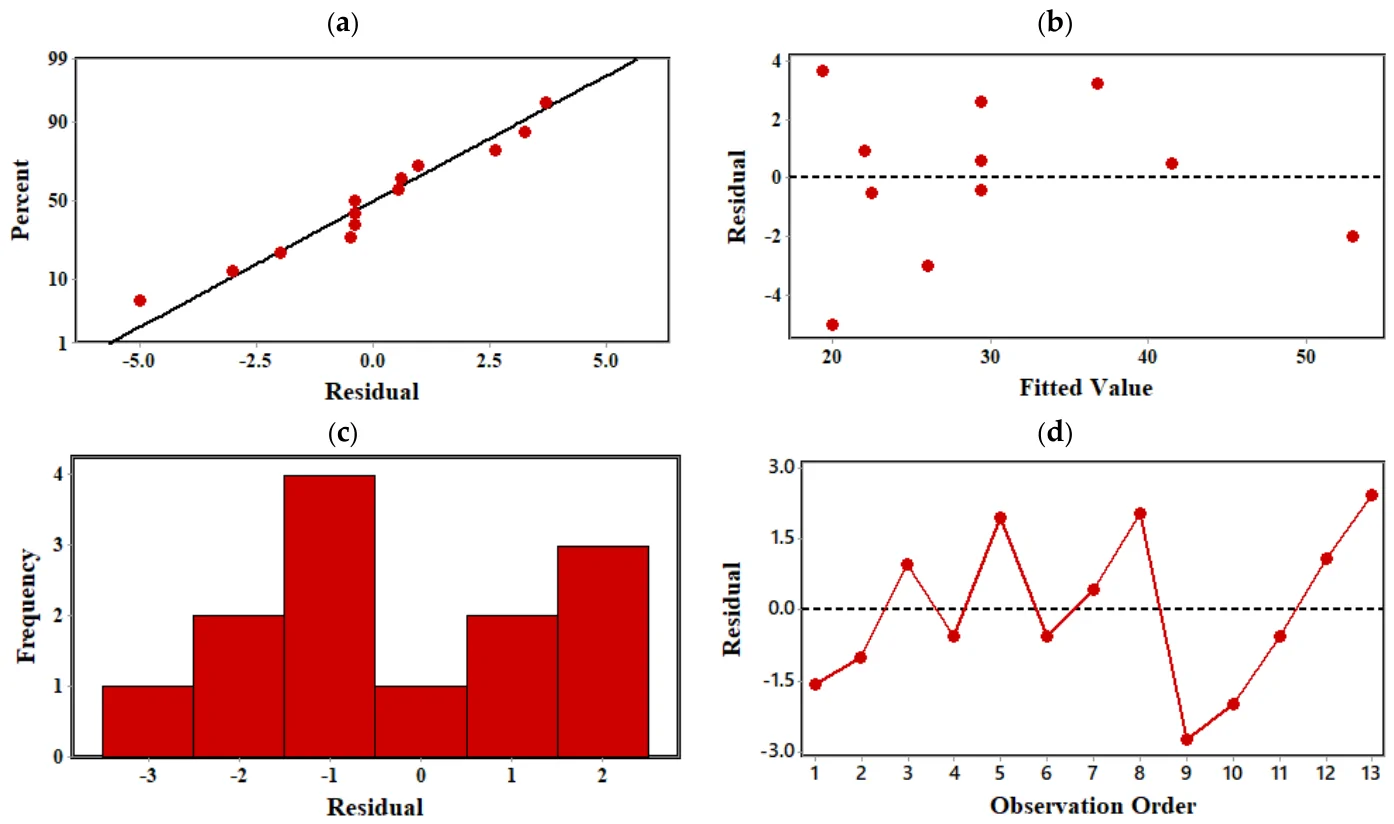
Analytic plots for apparent viscosity. (**a**) Normality plot; (**b**) residual error plot; (**c**) residual frequency bar-charts; and (**d**) residual at each run.

The influence of particle concentration on the apparent viscosity is shown in Figure 8a–d. The normality plot in Figure 8a shows that the data roughly lie along the straight line. This clearly indicates that the apparent viscosity data are normally distributed. The plot confirms no outliers in the dataset, as all points closely align with the straight line. This information indicates that there is no inconsistency amongst the experimental and model-predicted apparent viscosities. The fitting of the model was further evaluated using the residual error plot shown in Figure 8b. It is evident that the residuals were all randomly distributed around the zero mark with no clear pattern. This suggests that the model developed for apparent viscosity fits well with the experimental results. The residuals were plotted against the frequency, as shown in Figure 8c. The plot clearly shows that the residuals were approximately normally distributed with no skewness, kurtosis and outliers associated with the dataset. Moreover, the residual at each experimental run was also calculated and shown in Figure 8d. Seven out of 13 samples were close to the zero residual line, which indicates a stronger agreement between model and the experimental result. The maximum negative residual was associated with run 9, while the maximum positive was linked with run 13. Both of these runs contained equal concentrations of sodium alginate and nanocomposite material in the drilling mud.

### 3.8. Influence of Particles on Apparent Viscosity Through Interaction and Main Effect Plot

**Figure 9:**
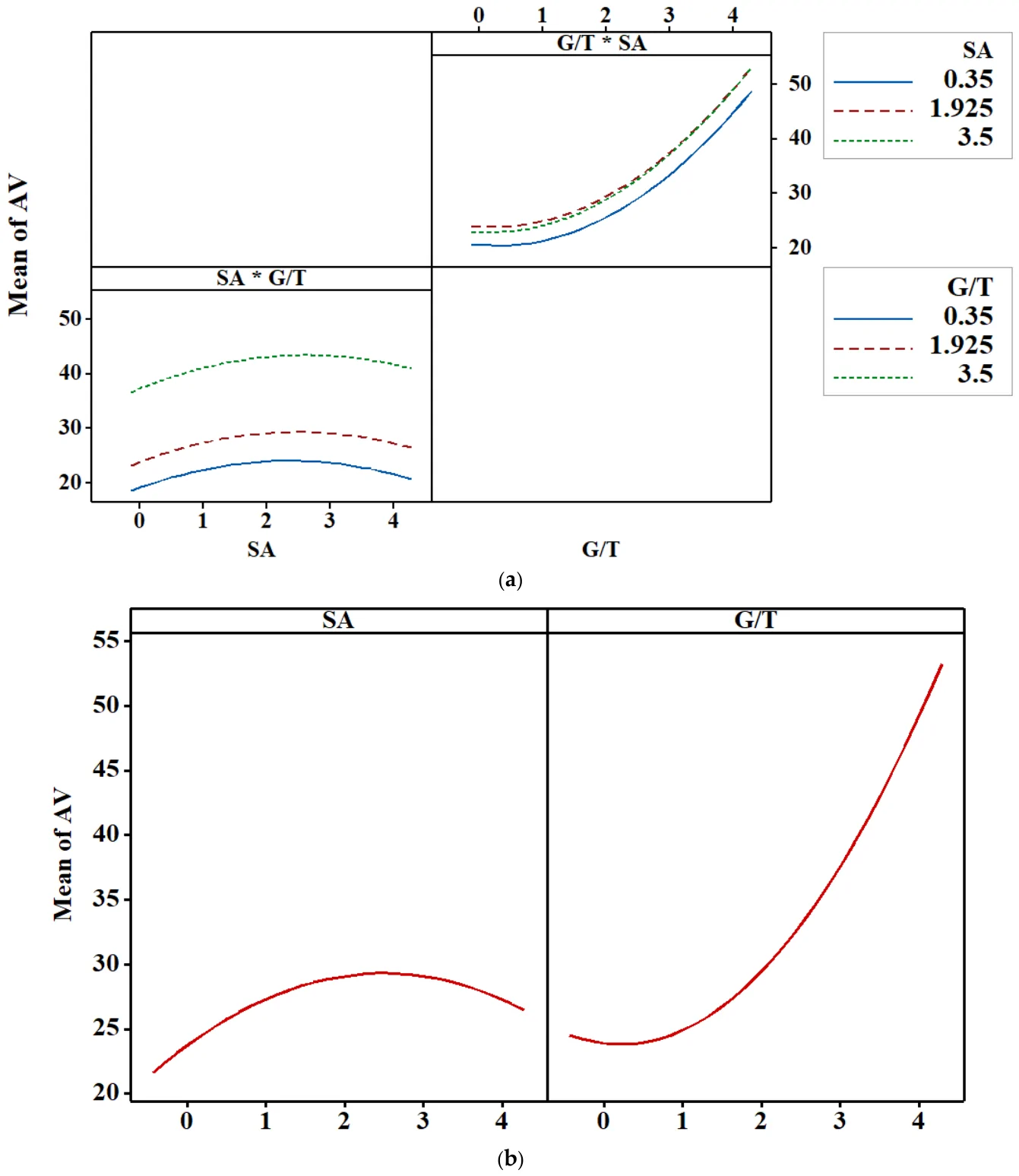
Analyzing the influence of particles on apparent viscosity using (**a**) interaction plot and (**b**) main effect plot (* means combined effect).

Figure 9 shows the influence of particles on apparent viscosity through the interaction and main effect plot. The interaction matrix, as shown in Figure 9a, clearly shows that, as the concentration of the nanocomposite increases, the impact on apparent viscosity also increases. When TiO_2_ combines with GO, the agglomeration of TiO_2_ decreases and hence improves the overall stability of the composite in the drilling mud. This eventually impacts the viscosity of the system. Moreover, these nanocomposite materials develop a network structure with sodium alginate through hydrogen bonding and increase the viscosity of the drilling mud. On the other hand, the impact on apparent viscosity is totally different when the concentration of sodium alginate changes in the mud system. A non-linear trend was observed in this case. At low concentrations of sodium alginate, there is a moderate increase in viscosity, which becomes significant at moderate concentrations due to the formation of robust hydrogen bonding and entanglement between the two entities. However, as the concentration increases above 3 g, the apparent viscosity reduces due to the dense polymer network, which creates clustering of the nanocomposite material. These trends are also similar to the main effect plot shown in Figure 9b.

### 3.9. Influence of Particle Concentration on Av Using 2D Contour and 3D Surface Plots

**Figure 10:**
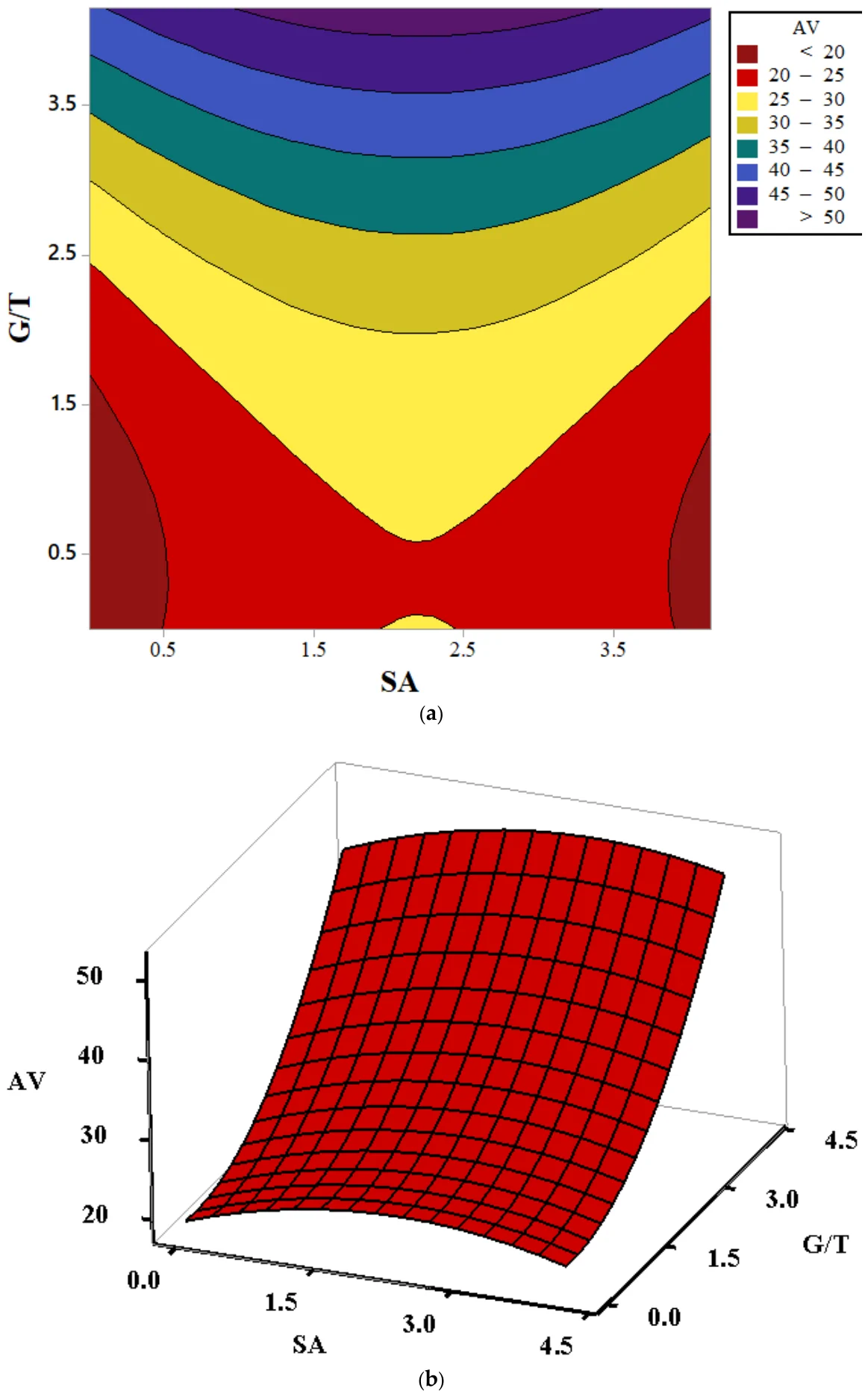
Influence of nanoparticles and sodium alginate on apparent viscosity through (**a**) contour plot and (**b**) surface plot.

Figure 10 shows the impact of particle concentration on the apparent viscosity using 2D contour and 3D surface plots. Both figures clearly show that, at low concentration, the particles had a minimum effect on the apparent viscosity. At low concentrations, biopolymer chains are sparse and distant, resulting in minimal entanglement and a weak structure. Consequently, the nanocomposites are inadequate to form an effective supporting network in the drilling mud. However, as the concentration of both entities increases in the drilling mud, the apparent viscosity also increases. A prominent increase in polymer–nanocomposite interactions was observed, which enhances the viscosity of the system as shown in Figure 10b. Additionally, the sodium alginate structure was strengthened by crosslinking between the polymer and the nanocomposite material. The most influential factor is the nanocomposite material, as shown by the pronounced increase in AV trend with nanomaterial addition in the surface 3D plot. These results also validate the observations shown in Figure 9b.

### 3.10. Apparent Viscosity Anova and Regression Model

A second-order predicted plastic viscosity equation, as shown by Equation (3), was developed in this article. The parameters ‘SA’ and ‘G/T’ represent the concentration of the polymeric material, sodium alginate, and the nanocomposite material: graphene oxide/titanium dioxide. The constants in Equation (2) are the adjustable parameters that show the best-fitted equation for the apparent viscosity. The result calculated from the model equation was statistically tested using the ANOVA analysis. Table 5 shows the result of the analysis of variance for the apparent viscosity model. The model demonstrated a statistically significant result, with a *p*-value 0.00 < 0.05. This clearly shows that there is a significant statistical relationship between the experimental and model-predicted apparent viscosity, and the null hypothesis was rejected. Furthermore, the nanocomposite material emerged as the most impactful additive, exhibiting a higher degree of statistical significance (*p*-value < 0.05) when compared with the polymeric material. The polymeric material initially increases the polymer–nanoparticle interactions through hydrogen bonding. Beyond this, the titanium dioxide forms hydrogen bonding with graphene oxide oxygenated groups. Furthermore, the structural complexity is increased due to the physical entanglement between GO sheets and sodium alginate chains and, finally, the nanomaterial reduces the slippage effect between the different fluid layers. All of these phenomena ultimately impact the apparent viscosity of the system. Additionally, the AV model developed in this study proves to be significant, with a *p*-value < 0.05 as shown in Table 5, while the lack of fit remained statistically insignificant, which clearly indicates that the newly developed regression model effectively describes AV experimental values. Furthermore, the coefficient of determination R^2^ = 96.97% shows that almost all of the experimental datasets can be properly explained through this model. The close agreement between R^2^ adjusted = 94.80% and R^2^ predicted = 85.04% further indicates the exceptional predictive capability without any indication of model overfitting.

(3) **Apparent Viscosity (cP)** = 16.68 + 7.68 ∗ SA − 1.31 ∗ G/T − 1.749 ∗ SA ∗ SA + 1.915 * G/T ∗ G/T + 0.000 ∗ SA ∗ G/T

### 3.11. Graphical Analysis of the Apparent Viscosity Predicted Model

**Figure 11:**
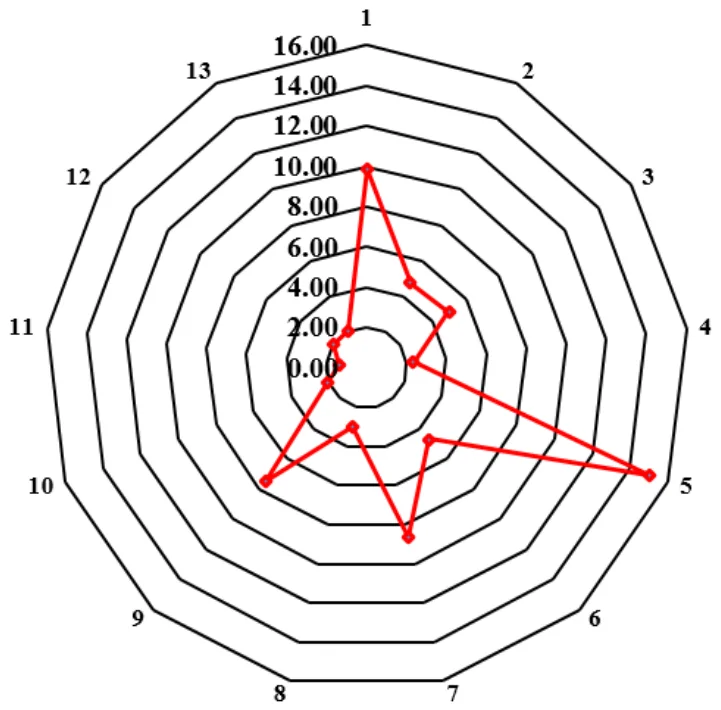
Graphical validation of the apparent viscosity regression model through radar error plot.

Figure 11 shows the graphical validation of the apparent viscosity regression model developed in Equation (3). The radar plot in Figure 11 demonstrates the model’s efficacy. The plot features relative errors of each experimental run. Out of 13 samples, 12 samples demonstrated an error level of less than 15%. The highest error was recorded at run 5, in which the concentration of biopolymer was 0 wt. %. Run 5 is part of the axial points shown in Table 2. These axial points in response surface methodology are situated at the edges of the experimental region. As a result, the process experiences more non-linear/unpredictable characteristics. The design of the experiment becomes more uncertain as fewer points exist at the edges. This gives rise to a high variance of prediction and ultimately increases the relative errors in this specific region. On the other hand, the minimum relative error was recorded in run 11, which is part of the center points, as shown in Table 2. These points are located at the center of the experimental region and are the most stable points. Hence, they exhibit a minimum variance of prediction and uncertainty, which eventually decreases the relative errors in these measurements. Table 6 shows the model-predicted apparent viscosities and the corresponding relative error percentages. It is evident that the relative error percentages of 12 out of 13 experimental runs is below 15%. Moreover, the maximum error was recorded in sample 5, which contains only nanocomposite material.

### 3.12. Technical and Economic Feasibility

All 13 mud samples tested in the design space were found to have an apparent viscosity greater than the minimum of 12 cP set by the API, and the regression models suggest that apparent viscosity and plastic viscosity can be adjusted in the range of 20–51 cP and 12–28 cP, respectively, for the tested design space through changes in the nanocomposite and sodium alginate loadings. The predicted plastic viscosity from the 0 to 4.15 g of the nanocomposite increased from 12.1 to 28.0 cP (+131%) while the apparent viscosity increased by 115% from 23.9 to 51.4 cP when the sodium alginate amount was fixed at 1.93 g. The quadratic models can be used to determine the minimum loading that produces the desired viscosity instead of the maximum loading tested, because high viscosity would increase pump pressure and the ECD. Standard API procedures were used to test the additives in the laboratory and no special equipment was required. All tests were conducted at ambient temperature and pressure, in a single bentonite–barite base fluid with no salt contamination, and no tests were conducted to assess the dispersion’s filtration or lubricity or to assess the long-term stability of the dispersion. The high-temperature, high-pressure and salty condition therefore needs to be tested prior to its use in the field.

The cost of a viscosity additive is important because it has been reported to be about 5–20% of the total cost of a well [52,53]. At the top of this design range, the amount of the nanocomposite added is 3.5 g/batch (approx. 10 kg/m^3^ of mud) and the sodium alginate added is 4.15 g (approx. 12 kg/m^3^ of mud). The uncertainty is mainly due to the cost of the nanocomposite. It is synthesized by batch hydrothermal synthesis (200 °C, 24 h) with graphene oxide and the synthesis of a TiO_2_-based nanocomposite has been reported as potentially cost prohibitive for large-scale drilling without optimization [54]. The high production cost of 2D nanomaterials in large quantities is also reported to make them more expensive than nanoparticle-free bentonite muds, but often there is less need for the 2D nanomaterials to produce the desired performance [52]. Xanthan gum is another common polymeric viscosifier, but is the most expensive of the additives in a recent review [53], and has a temperature- and brine-composition-dependent rheology [16].

The unit prices of the laboratory-synthesized materials are not representative of the bulk supply, so the options are compared by the viscosity that can be achieved per unit mass. The apparent viscosity of the models was increased by about 5.9 cP/g by increasing the nanocomposite from 0.35 g to 3.5 g and was increased by about 0.8 cP/g by increasing sodium alginate from 0.35 g to 3.5 g, while the plastic viscosity of models was increased by about 3.1 cP/g by increasing nanocomposite and decreased by about 0.1 cP/g by increasing sodium alginate. The delivered cost per kg of the nanocomposite is therefore considered to be less than the delivered cost per kg of the conventional product multiplied by the ratio of the viscosity gains per kg of the product, if the nanocomposite product is to be cost competitive with the conventional product. This ratio should be based on a conventional viscosifier control in the same base mud, not included in the present design. The cost–performance comparison of the above nanomaterials with commercial additives of this type has been reported [55]. A comparison between the proposed system and the conventional system is given in Table 7.

Sodium alginate is a renewable polysaccharide that has been described as eco-friendly and sustainable [46], whereas the environmental behavior, toxicity, and disposal of the TiO_2_–GO nanocomposite were not assessed in this study and should be evaluated together with scale-up of the synthesis and long-term stability before deployment.

## 4. Limitations

The study is based on the influence of nanocomposite material and biopolymer on the viscosities of the WBM. Nevertheless, there are still some limitations that require further action:

1. The long-term stability of the WBM containing nanocomposite material and biopolymer was not observed in the current study. Further investigation is required to evaluate dispersion stability, particle agglomeration and rheological consistency during prolonged storage and circulation.
2. Shale inhibition was not experimentally investigated in this study. Consequently, the efficacy of the developed formulations in mitigating shale swelling needs further investigation.
3. The current study is limited to laboratory scale evaluation under standard conditions. Field testing that replicates downhole conditions are required to confirm the practical applicability of the studied mud samples.

## 5. Conclusions

The article explores the effect of brown-algae-derived biopolymer and a hydrothermally synthesized nanocomposite material on the viscosities of drilling mud. The viscosity ultimately influences the cutting carrying and lifting capacity of the drilling mud. The following key conclusions were drawn from the study:

The concentration of nanocomposite material had a significant impact on increasing the rheological properties of the mud system.

The increase in viscosity is mainly due to the interaction between the oxygenated functional groups in graphene oxide and the sodium alginate polymer chain.

The high surface area and oxygen-bearing functional groups associated with the graphene oxide promote robust interaction with TiO_2_ particles. The composite tends to form a strong network structure, which ultimately enhances the viscosity of the drilling mud.

The experimental results and statistical analysis clearly indicate that the concentration of nanocomposite material significantly affects both viscosities. Nonetheless, the major quadratic influence of biopolymer on AV (*p*-value < 0.05) indicates that its roles are dependent on concentration and must not be ignored. These rheological characteristics are important for drilling fluid efficacy, particularly in wellbore cleaning, cutting removal and transportation. However, the degree to which the observed viscosity variations improve these operational functions needs additional confirmation under suitable drilling conditions.

## Figures and Tables

**Table 1:** Summary of representative previous studies on nanomaterial and biopolymer additives in water-based drilling fluids, and the position of the present work.

Study	Additive(s)	Main Findings	Limitations/Challenges
Kosynkin et al. (2012) [34]	Graphene oxide (GO)	GO acts as a high-performance fluid-loss-control additive in water-based mud.	Fluid loss is the target; viscosity is not optimized; no biopolymer or metal-oxide partner.
Sadeghalvaad and Sabbaghi (2015) [20]	TiO_2_/polyacrylamide nanocomposite	Lower filtration and higher mud viscosity.	Synthetic polymer matrix; no GO support and no biopolymer.
Al-Yasiri et al. (2019) [38]	Xanthan gum + SiO_2_	Higher yield point, lower filtrate loss, and better lubricity than conventional mud.	Spherical silica with xanthan; no carbon-supported composite; alginate not considered.
Rafieefar et al. (2021) [6]	GO	Rheology and filtration of GO muds studied by experiments, mechanistic analysis, and modeling.	GO alone; no TiO_2_ and no biopolymer.
Lalji et al. (2023) [37]	GO + pure-bore + sodium alginate (SA)	Factorial analysis of the effect on filtration properties.	Filtration is the response; no TiO_2_ nanocomposite; viscosity is not the main target.
Lalji et al. (2024) [35]	GO/TiO_2_ nanoparticles	Effects on rheology, filtration, and shale swelling evaluated by full factorial design.	No biopolymer; full factorial design rather than a response surface model.
Lalji et al. (2025) [31]	GO + rutile TiO_2_ (seawater mud)	Considerable fluid-loss reduction; TiO_2_ uniformly distributed over GO sheets.	Fluid loss is the focus; seawater mud only; no biopolymer.
Mozaffari et al. (2026) [39]	Silica, graphene, and zeolite nanoparticles (0.01–0.1 wt.%)	0.07 wt.% performed best; silica improved PV, YP, and gel strength, graphene reduced fluid loss.	Single nanoparticles in a narrow range; aggregation-induced degradation at 0.1 wt.%.
Shuheil, Alrhym et al. (2026) [40]	N,S-co-doped rGO/Cu_2_O nanocomposite	Optimum at 1000 ppm; API filtrate reduced by 58.8–61.3%; shale recovery increased by 20%.	Agglomeration above the optimum; hydrothermal synthesis may be costly to scale; no joint optimization with a second additive.
**This study**	TiO_2_–GO nanocomposite + SA	Central composite design (13 runs) gives predictive models for PV and AV; ANOVA shows the nanocomposite is the dominant factor.	Tested at standard temperature and pressure; HTHP and salinity effects remain for future work.

**Table 2:** Central composite design matrix defining the number of runs in the study.

Run	600 RPM Dial Reading ±0.5	300 RPM Dial Reading ±0.5	Sodium Alginate (g)	Graphene Oxide and Titanium Dioxide Composite (g)	Type of Run
1	46	36	0.35	0.35	**Factorial points**
2	46	36	3.5	0.35	
3	80	55	0.35	3.5	
4	84	56	3.5	3.5	
5	37	13	0	1.93	**Axial points**
6	44	26	4.15	1.93	
7	46	40	1.93	0	
8	102	77	1.93	4.15	
9	64	46	1.93	1.93	**Center points**
10	58	41	1.93	1.93	
11	60	45	1.93	1.93	
12	58	42	1.93	1.93	
13	58	43	1.93	1.93	

**Table 3:** Analysis of variance for the plastic viscosity model.

Source	DF	Adj SS	Adj MS	F-Value	*p*-Value
Model	5	483.105	96.621	36.39	0.000
Linear	2	465.915	232.957	87.73	0.000
SA	1	1.288	1.288	0.49	0.509
G/T	1	464.467	464.467	174.92	0.000
Square	2	13.345	6.673	2.51	0.150
SA*SA	1	12.058	12.058	4.54	0.071
G/T*G/T	1	0.917	0.917	0.35	0.575
Two-way interaction	1	2.250	2.250	0.85	0.388
SA*G/T	1	2.250	2.250	0.85	0.388
Error	7	18.587	2.655		
Lack-of-fit	3	11.787	3.929	2.31	0.218
Pure Error	4	6.800	1.700		
Total	12	501.692			

**Table 4:** Model-predicted plastic viscosity and relative error %.

Runs	SA (g)	G/T (g)	PV ± 0.7	RSM Model PV	R.E. %
1	0.35	0.35	10	9.55	4.49
2	3.5	0.35	10	8.88	11.22
3	0.35	3.5	25	23.95	4.22
4	3.5	3.5	28	26.27	6.18
5	0	1.93	24	17.79	25.88
6	4.15	1.93	18	19.61	8.93
7	1.93	0	6	5.81	3.22
8	1.93	4.15	25	26.55	6.21
9	1.93	1.93	18	16.14	10.33
10	1.93	1.93	17	16.14	5.06
11	1.93	1.93	15	16.14	7.60
12	1.93	1.93	16	16.14	0.88
13	1.93	1.93	15	16.14	7.60

**Table 5:** Analysis of variance for the apparent viscosity model.

Source	DF	Adj SS	Adj MS	F-Value	*p*-Value
Model	5	1034.85	206.971	44.75	0.00
Linear	2	688.04	344.021	74.38	0.00
SA	1	16.36	16.359	3.54	0.102
G/T	1	671.01	671.013	14.08	0.000
Square	2	252.35	126.174	27.28	0.000
SA*SA	1	108.93	108.934	23.55	0.002
G/T*G/T	1	130.70	130.702	28.26	0.001
Two-way interaction	1	0.00	0.00	0.00	1.000
SA*G/T	1	0.00	0.00	0.00	1.000
Error	7	32.38	4.625		
Lack-of-fit	3	25.58	8.525	5.01	0.077
Pure Error	4	6.80	1.700		
Total	12	1067.23			

**Table 6:** Model-predicted apparent viscosity and relative error %.

Runs	SA (g)	G/T (g)	AV ± 0.25	RSM Model AV	R.E. %
1	0.35	0.35	21	18.93	9.86
2	3.5	0.35	23	21.91	4.74
3	0.35	3.5	40	38.03	4.93
4	3.5	3.5	42	41.01	2.36
5	0	1.93	18.5	21.28	15.05
6	4.15	1.93	22	23.03	4.70
7	1.93	0	23	24.99	8.64
8	1.93	4.15	51	52.53	3.00
9	1.93	1.93	32	29.59	7.52
10	1.93	1.93	29	29.59	2.04
11	1.93	1.93	30	29.59	1.36
12	1.93	1.93	29	29.59	2.04
13	1.93	1.93	29	29.59	2.04

**Table 7:** Comparison of the proposed system with conventional viscosity-control options.

Additive	Technical Assessment	Cost and Practical Constraints
**Bentonite (base viscosifier)**	Provides the baseline viscosity and filter-cake solids in the base mud used here [44].	Used at 65 g per batch; the additives in this study act on top of this baseline.
**Xanthan gum**	Widely used polymeric viscosifier; its rheology depends on temperature and brine composition [16].	Highest unit cost among the common additives compared by Mahmoud et al. [53]
**Na-CMC and PAC-LV**	Sodium alginate maintained rheology and filtration better than both under salt contamination [45].	Not costed in the present study.
**Sodium alginate alone (this study)**	Small effect: about +0.8 cP AV and −0.1 cP PV per gram (model, 0.35–3.5 g at 0.35 g nanocomposite).	Renewable biopolymer; up to 4.15 g per batch (about 12 kg per m^3^).
**TiO_2_–GO nanocomposite + sodium alginate (this study)**	Model-predicted PV 12–28 cP and AV 20–51 cP; about +5.9 cP AV and +3.1 cP PV per gram of nanocomposite.	Batch hydrothermal synthesis and GO cost uncertain at scale; tested at ambient conditions only.

AV: apparent viscosity; PV: plastic viscosity. Per-gram values are model predictions from Equations (4) and (5).

## Data Availability

All data generated or analyzed during this study are included in this published article.
